# New Light on the Afferent Nervous System

**Published:** 1905-12-23

**Authors:** 


### Hospital Clinics.

### NEW LIGHT ON THE AFFERENT NERVOUS SYSTEM.

The summer number of "Brain" is of quite unusual interest, for which the world of medicine is indebted to the enterprise and self-sacrifice of Dr. Henry Head. For two years careful observations were carried out upon patients admitted to the London Hospital for injuries to the peripheral nerves, with the result that certain conclusions were achieved which appeared to be at variance with teachings founded upon the anatomical distribution of the afferent nerves. For instance, when the median nerve is divided, sensation is entirely lost over a large part of both the index and middle fingers; while over the area of the palm credited by anatomists to the influence of the median nerve, sensation is usually diminished but not abolished. Similarly, division of the ulnar nerve produces complete insensibility of the little finger, and of a variable portion of the ulnar aspect of the hand, while partial loss of sensation is found over a larger area of the palm and over the ulnar aspect of the ring-finger. This area of diminished sensibility is in reality an area in which some kinds of sensibility are lost and others retained. Thus it is insensitive to certain higher forms of stimulation such as the light touch produced by carded cotton wool; over it there is no appreciation of temperatures between 22° C. and 40° C.; lastly, over it it is impossible to identify the double character of a double stimulus such as is supplied by a pair of compasses. When the shock of the injury has passed off the area of diminished sensibility which, as has been said above, is insensitive to higher forms of stimulation, becomes endowed with a new responsiveness to stimuli producing pain. The prick of a pin now causes a more unpleasant effect over the area of diminished sensibility than it does over normal areas of skin.

If the divided nerve be united, sensation begins to return after a variable interval of time, the first sign of recovery being a gradual diminution in the size of the area insensitive to pain and to all degrees of heat and cold. But departure of the analgesia does not necessarily connote the reappearance of sensibility to the higher forms of stimulation. In a hand so far recovered that sensibility to pain, heat, and cold has vastly improved, the line of demarcation between areas responsive and not responsive to light touch may be as clearly defined as it was in the days immediately following the injury; and this condition may persist for months. Further, in a hand so far recovered as to be sensitive to pain and temperature over areas previously insensitive to them, this sensibility is strangely altered. A prick is appreciated, but produces a sensation which radiates widely over the affected area. It causes unnatural discomfort, and the patient has an uncontrollable desire to withdraw his hand. Moreover, although ice, and water at a temperature of 50° C. are appreciated as cold and hot respectively, intermediate degrees produce no sensation of temperature, and water at 25° C. or 26° C. may be indistinguishable from water at 40° C.

Having assured themselves of the truth of these conclusions by clinical observation upon patients accidentally presenting themselves, Dr. Head and his collaborators determined that to obtain an exhaustive account of the sensory condition of parts robbed of their nerve-supply it was necessary that the patient should be a trained observer, and the injury determined beforehand. Therefore Dr. Head submitted himself to an operation by which his radial and his external cutaneous nerves were divided in the neighbourhood of the elbow. Small portions of each were excised, and the ends united with silk sutures. "This operation produced loss of all forms of cutaneous sensation over an extensive area on the radial half of the forearm and back of the hand. Stimulation with cotton-wool, the prick of a pin, the application of all forms of heat and cold, were unappreciated, and the two points of a pair of compasses could not be discriminated even when separated to the furthest extent possible. But if this part was touched with the point of a pencil, the head of a pin, or even the ball of the finger, the stimulus was at once appreciated, and the point of application localised with remarkable accuracy. We are thus face to face with the conclusion that complete destruction of all sensory nerves to the skin leaves the part sensitive to most of those stimuli commonly used by the physician and surgeon as a test of sensibility to touch."

This remarkable experiment has demonstrated conclusively that the fibres subserving deep sensibility run with the motor nerves, though themselves afferent. Sherrington has traced them to muscles, tendons, and joints, but, as Dr. Head observes, the operation on his arm gave him and his assistants a unique opportunity of exposing to numerous tests a part endowed with deep sensibility alone.

The peculiarity of a part in this condition, that is to say, the peculiar aptitude of the afferent fibres of a muscular nerve, is the appreciation of stimuli which produce change of form. Thus pressure or jarring of the skin were easily appreciated, but elevation of the skin by tension upon a hair produced no effect upon consciousness. This experiment proves, incidentally, that the sensibility to pressure was not due to any residual afferent fibres of an ordinary kind. All sense of form and size was lost; excess of pressure produced aching pain, but the affected parts could be burnt or exposed to a temperature below the freezing point without giving rise to sensations of pain, or heat, or cold.

Seven weeks later sensation to prick began to return to the arm, and thirteen weeks after the operation there was no part of the forearm upon which a prick could not be appreciated. Yet for more than a year these parts remained completely insensitive to light touch, and even now, more than two years after the operation, the back of the hand has not entirely recovered its sensibility to such fine stimuli. It was thus possible to test exhaustively a part sensitive to prick but insensitive to the lightest touch. In such a part the pain produced by a painful stimulus does not remain localised to the point of application, but radiates widely, and may even be referred to parts quite remote. Ice was appreciated as a cold body, and water at a temperature of 50° C. as a hot body, but intermediate degrees produced no effect upon consciousness. This peculiarity was found to be due to the presence of "hot spots" and "cold spots." It was possible to demonstrate at one period of recovery, discrete areas at which alone sensations of cold could be appreciated. These spots could be stimulated by any temperature below about 24° C. The "hot spots," which were scantier, reacted to temperatures whose lower limit varied from 38° to 45° C. Stimulation of these spots resulted in sensations of heat and cold of wider distribution than the area stimulated appeared to warrant. Similar radiation was observed in the returning sensations of pain, the radiation being not fortuitous but constant in its distribution for any given stimulus and stimulated area. Lastly, in this stage of recovery the hairs manifest a peculiar sensibility. Movement of a hair upon the normal skin produces a sensation of touch which is exceedingly well localised; but under the conditions of recovery after nerve-section with which we are at present concerned, movement of a hair produces a widespread tingling.

To the sensibility described in the last paragraph, namely, that of a part which has recovered its sensibility to prick, but remains void of sensibility to light touch, Dr. Head has given the name *protopathic*. The return of protopathic sensibility brings a cessation of all the destructive changes which are liable to occur on an insensitive skin.

After a variable interval the affected part begins to be sensitive to light touch, and to the degrees of temperature which produce the sensations called "warm" and "cool" on the normal skin. Localisation and the appreciation of doubleness in stimuli become progressively established, and the skin resumes its normal functions. To this sensibility to the higher forms of stimulation Dr. Head has given the name *epicritic*.

It is thus clear that there are two varieties of sensation, the protopathic and the epicritic, supplied to all areas of the skin. By a curious chance in the matter of distribution of the fibres subserving these two varieties, Dr. Head was able in his own case to demonstrate the fact that these fibres were not all modifications of one and the same system. He found that over the radial half of the dorsum of his wrist was a small area over which protopathic sensibility was lost while epicritic sensibility remained. This left it a matter past all doubt that these two varieties of sensation depended upon separate structures. Further, it is clear that these two systems are regenerated after injury with a very unequal facility, the protopathic system recovering its functions long before the epicritic system.

Every peripheral nerve contains in varying proportions the fibres which subserve these two functions. Firstly as regards the epicritic supply: If the hand and forearm be divided by a continuation of the line which forms the long axis of the ring-finger, the hand and forearm will be divided into a pre-axial portion which includes the thumb, and a post-axial portion which includes the little finger. The post-axial portion is supplied by the ulnar and internal cutaneous nerves; the pre-axial by the median, the radial, the external cutaneous, and the long branch of the musculo-spiral. If, therefore, the peripheral nerves of this region are gathered into certain groups, overlapping of epicritic sensation depending upon these groups is very slight. These groups are as follows: (1) Ulnar and internal cutaneous; (2) median; (3) the remainder of the pre-axial group. But in the case of protopathic sensibility enormous overlapping occurs. The outstanding difference in the distribution of the fibres subserving the two systems lies in the fact that the unit of epicritic supply is the peripheral nerve, the unit of protopathic supply is the posterior root.

The sensory mechanism of the peripheral nerves is thus found to consist of three systems:—

1. Deep sensibility, capable of answering to pressure and the movement of parts. The fibres subserving this form of sensation run with the motor nerves and are not destroyed by division of all the sensory nerves to the skin.

2. Protopathic sensibility, capable of responding to painful cutaneous stimuli, and to the extremes of heat and cold. This is the great reflex system, producing a rapid and widely diffused response unaccompanied by any definite appreciation of the spot stimulated.

3. Epicritic sensibility, by which we gain the power of cutaneous localisation, of the discrimination of two points, and of the finer gradations of temperature.

Dr. Head's experiments upon the sensibility of the gut, conducted upon patients upon whom the operation of colotomy has been performed, leads him to the view that the sensibility of the intestines is of a kind very similar to that which he has named protopathic. If this be so, it is, as he says, no adventurous guess to suppose that the protopathic system of the skin is one with the afferent sympathetic supply of the viscera. The new conception of the nature of the afferent fibres in nerves may therefore be summed up as follows:

The whole body, within and without, is supplied by the protopathic system, the somatic fibres supplying the skin, the visceral fibres supplying the internal organs. Another set of afferent fibres, peculiarly associated with impulses of movement or pressure, exist in connection with the Pacinian organs. These fibres run in conjunction with the motor nerves. Finally, there is the epicritic system of fibres with which the surface of the body only is supplied. This system endows the skin with sensibility to light touch, with capacity to discriminate the doubleness of stimuli and minor degrees of heat and cold. The fibres of this system are the most easily injured, and regenerate after injury far more slowly than do those of the protopathic system. They are evidently more highly developed and approach in the time required for their regeneration the motor fibres supplied to voluntary muscle.

